# Intratumoral *Leptotrichia* is a novel microbial marker for favorable clinical outcomes in head and neck cancer patients

**DOI:** 10.1002/mco2.344

**Published:** 2023-08-27

**Authors:** Shuting Yu, Junru Chen, Fangxu Yan, Xingming Chen, Yan Zhao, Peng Zhang

**Affiliations:** ^1^ Department of Otolaryngology‐Head and Neck Surgery, Peking Union Medical College Hospital Peking Union Medical College and Chinese Academy of Medical Sciences Beijing China; ^2^ Institute of Chinese Medical Sciences University of Macau Taipa Macao China; ^3^ Department of Otolaryngology Head and Neck Surgery, Beijing TonGren Hospital Capital Medical University Beijing China; ^4^ Beijing Key Laboratory for Genetics of Birth Defects, Beijing Pediatric Research Institute, MOE Key Laboratory of Major Diseases in Children, Rare Disease Center, Beijing Children's Hospital, Capital Medical University, National Center for Children's Health Beijing China

Dear editor:

Head and neck squamous cell carcinoma (HNSCC) accounts for more than 70 million cancer cases annually around the world.^1^ The pathogenesis of HNSCC is not fully understood, but several risk factors for HNSCC, such as smoking, alcohol consumption, and human papillomavirus infection, have been reported. Recently, studies of the microbiomes of patients with head and neck cancer suggest that microbial variation correlates with the development of HNSCC, and some potentially oncogenic bacteria have been identified.^2^ However, there are limited reports exploring the microbiomes of patients with different stages of HNSCC. Data from other tumors demonstrate that microbial‐host factors, independent of the genomic composition of the tumor, may determine tumor behavior and patient outcomes; therefore, we hypothesized that patients with different stages of HNSCC would have diverse microbial compositions. In this study, we sought to identify microbial differences between early‐stage (T1–T2) and advanced‐stage (T3–T4) HNSCC patients.

16S ribosomal RNA sequencing data and clinical profiles of HNSCC patients were obtained from the Cancer Genome Atlas (TCGA) and the Cancer Microbiome Atlas (TCMA) database as described in the Supporting Information. There were total of 153 patients (T1–T2, *N* = 61; T3–T4, *N* = 92) included in this study from the TCGA database (Table S1). To measure whether the microbial composition was different between normal tissues, early‐stage tumor tissues, and advanced‐stage tumor tissues, we used principal coordinate analysis (PCoA) and permutational multivariate analysis of variance (PERMANOVA) based on the Bray‒Curtis distance from the genus profile. The results presented a significantly different distribution between normal tissues and tumor tissues (normal vs. T1–T2, *p*‐value = 0.035; normal vs. T3–T4, *p*‐value = 0.004) (Figure 1A). Then, differences in alpha‐diversity indices, including Chao1 index and Shannon index, were measured (Figure 1B). The results showed that the microbial alpha‐diversity was significantly decreased in T3–T4 samples compared with normal samples, and in T3–T4 samples compared with T1–T2 samples. Mucosal sites in the head and neck region harbor a site‐specific microbiome, which has an essential role in maintaining health and homeostasis. As high microbial diversity is considered to be a sign of health, and the microbiome is characterized by an overgrowth of diverse bacteria, such as *Fusobacterium, Prevotella, Veillonella*, and so forth, in head and neck malignancies, the decrease in alpha‐diversity of T3–T4 samples was consistent with previous studies. We next sought to examine tissue microbiome composition in three groups. At the phylum level, the tissue microbiota was dominated by members of Bacteroidetes and Firmicutes, followed by Fusobacteria (Figure 1C), which were similar to those reported in previous studies.^2^ We then compared the relative abundance of the top 20 genera in the three groups, which revealed the enrichment of specific genera, such as *Fusobacterium*, *Treponema*, and *Capnocytophaga*, in T1–T2 and T3–T4 samples compared with normal samples (Figure 1D).

**FIGURE 1:**
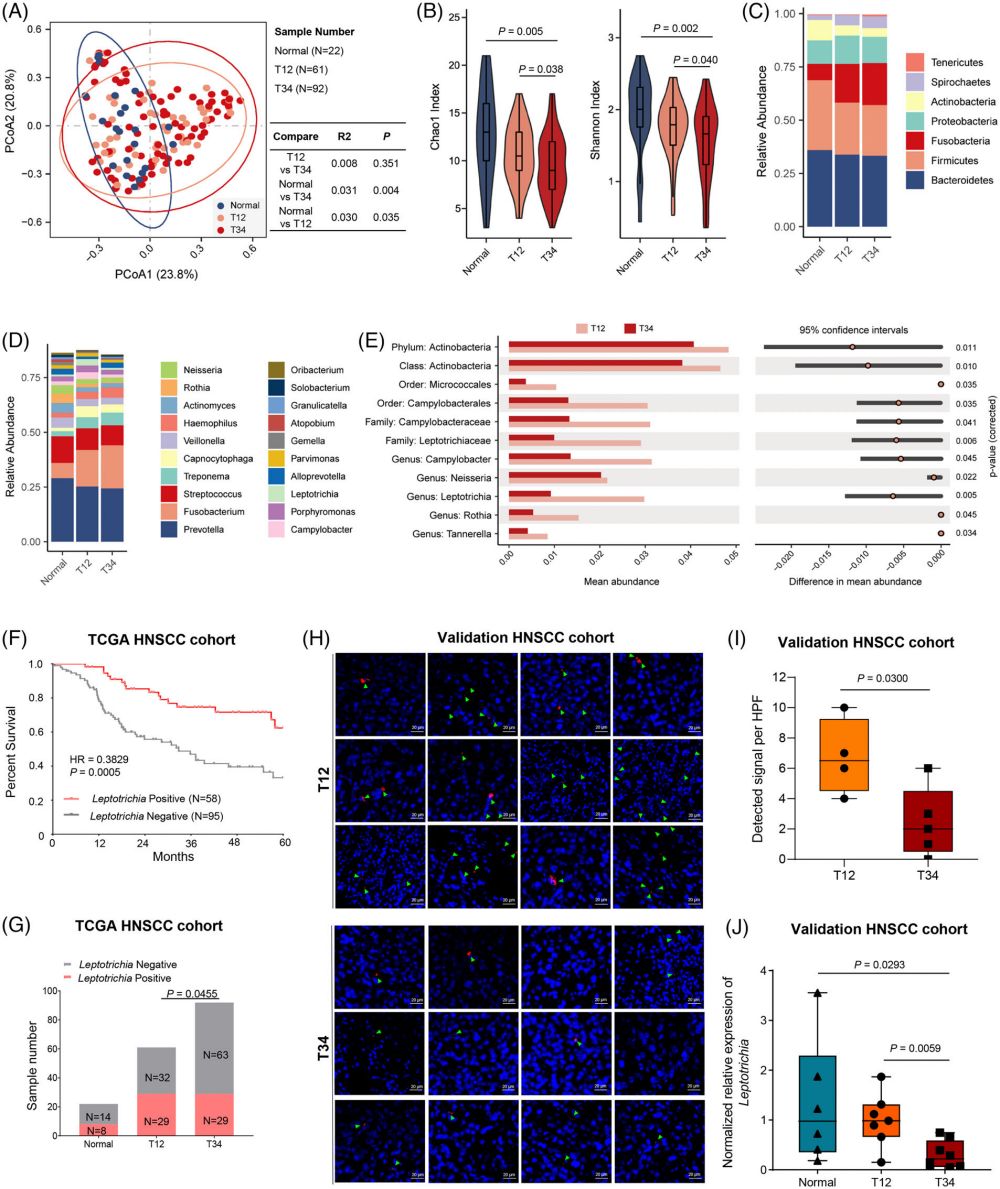
The association of *Leptotrichia* with head and neck squamous cell carcinoma (HNSCC) stages and patient outcomes. (A) Beta‐diversity calculated by principal coordinate analysis (PCoA) plots. The *p*‐value was derived from permutational multivariate analysis of variance (PERMANOVA). (B) Comparison of microbial alpha‐diversity among groups. *p*‐Values were calculated from the Wilcoxon rank‐sum test. *p* < 0.01 was labeled with “+” and “*” represented *p* < 0.05. (C) The relative abundance of dominant phyla in different groups. (D) The relative abundance of the top 20 dominant genera in different groups. (E) Significant differences in tumor microbial communities between T1–T2 tissues and T3–T4 tissues. (F) Overall survival of HNSCC patients based on whether *Leptotrichia* was detected in tumor samples based on the TCGA dataset. (G) Detection rate of *Leptotrichia* in different stages of HNSCC based on the TCGA dataset. (H) Fluorescence in situ hybridization (FISH) of tumor tissues from stage T1–T2 HNSCC or stage T3–T4 HNSCC. *Leptotrichia* genomic DNA was used as a probe (red) for FISH. The arrows indicate FISH signals. Scale bars: 20 μm. (I) Detected FISH signal per high‐power field (HPF) of samples. (J) The relative expression level of *Leptotrichia* genomic DNA in tumor tissues and paired normal tissues from stage T1–T2 HNSCC or stage T3–T4 HNSCC patients.

To extend our understanding of the role of the microbiome and its association with tumor stage and patient outcomes, we compared the relative abundances of bacterial taxa between T1–T2 tumor tissues and T3–T4 tumor tissues, finding significant differences in one phylum, one class, two orders, two families, and five genera (Figure 1E). Compared with samples from advanced‐stage (T3–T4) patients, samples from early‐stage (T1–T2) patients had significantly increased mean abundances of the genera *Campylobacter*, *Neisseria*, *Rothia*, *Leptotrichia*, and *Tannerella*. The parent taxa of the genus *Rothia* were also significantly increased in early‐stage patients compared with advanced‐stage patients, up to the phylum level. *Campylobacter* spp. were identified as strongly associated and enriched in colorectal cancer tissues, but their role in HNSCC remains controversial. *Neisseria* spp. and *Rothia* spp. were associated with a decreased risk of developing HNSCC^3^; in addition, depletions of *Neisseria* and *Rothia* in HNSCC cases were associated with worse cancer‐specific survival. *Tannerella* spp. was reported to be less abundant in HNSCC patients than in normal controls, despite their positive correlation with grade 2+ radiation‐induced oral mucositis.^3^, ^4^

To further investigate these findings, we tested the relationship between tumor microbial composition and overall survival (OS) in the HNSCC cohort by stratifying the patients into two groups based on whether the above five genera could be detected in tumor samples. Kaplan‒Meier curves were plotted for survival distributions (Figure 1F). The median OS times of the *Leptotrichia‐*positive and *Leptotrichia*‐negative groups were 37.5 and 20.5 months, respectively. Patients with *Leptotrichia‐*positive samples had significantly prolonged OS compared with those with *Leptotrichia‐*negative samples (*p*‐value = 0.0005) using univariate Cox proportional hazard models (Figure 1F). To decrease the influence of confounding factors, we performed a multivariate analysis based on basic characteristics and survival data of the TCGA cohort. *Leptotrichia* was found to be an independent factor of OS and was correlated with better OS (Table S2). In addition, the proportion of *Leptotrichia‐*positive samples in early‐stage (T1–T2) patients was approximately 1.5‐fold higher than that in advanced‐stage (T3–T4) patients, which means *Leptotrichia* was more prevalent in samples from early‐stage HNSCC patients than in samples from advanced‐stage patients (Figure 1G). These results suggest that *Leptotrichia* may have a protective effect in the HNSCC tumor microenvironment; therefore, we conducted fluorescence in situ hybridization (FISH) and quantitative PCR (qPCR) in HNSCC samples from patients in Peking Union Medical College Hospital to detect the genomic DNA of *Leptotrichia*. Using *Leptotrichia* genomic DNA as probes, we detected fluorescence signals in HNSCC tumor slides (Figure 1H). By calculating signals per high‐power field (HPF) in each sample, more FISH signals were identified in samples from stage T1–T2 patients than in samples from stage T3–T4 patients (Figure 1I). As fluorescence signals were relatively sparse in these samples, qPCR experiments based on a total of 15 HNSCC tumor samples and six matched normal samples were applied to better quantify the relative expression level of *Leptotrichia*. The results showed that the gene expression level of samples from stage T1–T2 patients was significantly higher (*p*‐value = 0.0059) than those in samples from stage T3–T4 patients (Figure 1J). Additional analysis of the composition difference of microbiome in different locations of head and neck cancers is shown in Table S3.

Several studies have reported that *Leptotrichia* along with *Fusobacterium* might promote the development of colorectal cancer, and *Leptotrichia* is significantly enriched in early‐stage colorectal cancer compared with late‐stage colorectal cancer.^5^ However, as a common bacterial genus in a healthy oral cavity, the role of *Leptotrichia* in HNSCC has rarely been reported in the literature. *Leptotrichia* may exert a protective role against malignancy in the upper respiratory tract by inducing the host cellular immune response, which needs further exploration.

In summary, our results demonstrated that the microbial composition of early‐stage HNSCC tumor tissues was distinct from that of advanced‐stage HNSCC tumor tissues, indicating a probable microbial effect on tumor behavior. In addition, we discovered that *Leptotrichia* was significantly increased in early‐stage patients compared to advanced‐stage patients and was correlated with better OS, suggesting a protective value of *Leptotrichia* in the HNSCC tumor microenvironment. Our study provided a strong rationale for further investigations to validate these discoveries and to extend these initial findings for the potential clinical development of microbiota‐based cancer therapies.

## AUTHOR CONTRIBUTIONS

P.Z., Y.Z., and X.C. provided the overall study design and co‐wrote the paper. S.Y. designed the study, collected samples, and drafted the paper. J.C. was responsible for the data analysis and co‐writing of the manuscript with the help of F.Y. All authors approved the final version of the manuscript.

## CONFLICT OF INTEREST STATEMENT

The authors declare they have no conflicts of interest.

## FUNDING INFORMATION

This study is supported by National High Level Hospital Clinical Research Funding (2022‐PUMCH‐B‐094), CAMS Innovation Fund for Medical Sciences (CIFMS) (2021‐I2M‐1‐023/2020‐I2M‐2‐009), and Science & Technology Fundamental Resources Investigation Program (Grant No. 2022FY100800).

## ETHICS STATEMENT

The study was conducted in accordance with the Declaration of Helsinki (as revised in 2013). The current study was approved by the Ethics Committee of Peking Union Medical College Hospital (approval ID number: ZS‐3148), Peking Union Medical College, and the Chinese Academy of Medical Sciences, Beijing, China. Written informed consent was obtained from all patients before enrollment.

## Supporting information

Supporting Information

Supporting Information

## Data Availability

The patient cohort materials used for the current study are publicly available and can be accessed from the TCGA database (https://portal.gdc.cancer.gov/, https://www.cbioportal.org/). The processed data and analysis codes are available upon reasonable request from the corresponding author.
