# Supplementary material for: Intratumoral Leptotrichia is a novel microbial marker for favorable clinical outcomes in head and neck cancer patients
Source: MedComm (2020). 2023 Aug 27;4(5):e344. doi: 10.1002/mco2.344 (PMC10460933; doi:10.1002/mco2.344)
Supplement: Supplementary file 1 — Supporting Information [file MCO2-4-e344-s001.docx]

**Supplementary Information for**

**Intratumoral *Leptotrichia* is a novel microbial marker for favorable clinical outcomes in head and neck cancer patients**

**Shuting Yu^1#^, Junru Chen^2#^, Fangxu Yan^1^, Xingming Chen^1^*, Yan Zhao^3^*, and Peng Zhang^4^***

^1^Department of Otolaryngology-Head and Neck Surgery, Peking Union Medical College Hospital, Peking Union Medical College and Chinese Academy of Medical Sciences, Beijing, China

^2^Institute of Chinese Medical Sciences, University of Macau, Taipa, Macao, China

^3^Beijing Tongren Hospital, Capital Medical University, Beijing, China

^4^Beijing Pediatric Research Institute, Beijing Children's Hospital, Capital Medical University, National Center for Children's Health, Beijing, CHINA

**^#^** Contributed equally

*Correspondence should be addressed to

Peng Zhang Ph.D., Professor. E-mail: zhangpengdyx@163.com

Yan Zhao Ph.D., Associate Professor. E-mail: zhaoyanray@126.com

Xingming Chen M.D., Professor. E-mail: xingming.chen@hotmail.com

**Supplementary Methods**

**TCGA patient cohort**

16S ribosomal RNA sequencing data and clinical profiles of HNSCC patients were obtained from the Cancer Genome Atlas (TCGA) database (http://cancergenome.nih.gov/, February 2021) and The Cancer Microbiome Atlas (TCMA) as described ^1,2^.

**Validation HNSCC patient cohort**

Patients were enrolled in this study at Peking Union Medical College Hospital (PUMCH) in 2022. All patients had pathologically confirmed, previously untreated HNSCC, and underwent radical resection. The exclusion criteria were as follows: (i) antibiotic therapy in the previous month; (ii) infection with HBV, HCV, syphilis, or HIV; and (iii) a history of malignant tumors, chemotherapy, or radio therapy. The TNM stages of all participants were identified according to the National Comprehensive Cancer Network (NCCN) Guidelines in 2021. Before sampling, participants were banned from dieting, smoking, and oral hygiene prophylaxis for at least 2 hours. Cancerous tissues from the central area of the lesions were obtained during surgery. Tissue samples were placed into sterile 2 mL Eppendorf tubes (Axygen, USA) and then frozen at −80°C before further processing.

**Statistics**

Alpha-diversity, based on the number of observed species, chao1 index, and Shannon index, and Beta-diversity, based on the pairwise bray-Curtis distance among samples, were calculated with R software (version 4.0.2). Differences in the Chao1 index, Shannon index, and observed species were detected using the Wilcoxon rank sum test. Principal coordinate analysis (PCoA) was conducted using the Analysis of Phylogenetics and Evolution (APE) package in R software^3^. PERMANOVA was performed with the vegan package in R to clarify differences in microbial communities between groups. The Wilcoxon rank sum test was applied to identify significantly different taxa between groups, and a false discovery rate <0.05 was considered to be statistically significant. Kaplan‒Meier curves were plotted for survival distributions with SPSS version 23.0 (IBM Corporation, Armonk, NY, USA).

**Microbial DNA extraction and *Leptotrichia* quantification**

Tumor tissues of HNSCC patients were divided into small pieces and digested in phosphate-buffered saline containing an enzymatic cocktail for 1 h at 37 °C ^4^. A 200 μl aliquot from each tissue was processed using QIAamp DNA Mini Kit (QIAGEN) according to the manufacturer’s instructions for the extraction of total genomic DNA. qPCR was performed to detect the *Leptotrichia* level by using 20 ng genomic DNA in 10 μl universal SYBR Green PCR Master Mix (Invitrogen, Thermo Fisher, Grand Island, NY, USA) with a StepOne Plus Real-Time PCR system (Applied Biosystems, Thermo Fisher, Grand Island, NY, USA). Gene ABC transporter of Leptotrichia was used in PCR. *Leptotrichia* quantitation was measured relative to the *Actb* gene. The *Leptotrichia* forward primer was 5′-GTTAAATGGGCTTCTTAAACCAA-3’; the reverse primer was 5’-AACAACTGTAAAATTGCCTT-3’.

**Fluorescence in situ hybridization (FISH)**

The tumor tissues of HNSCC patients were collected from frozen sections for in situ hybridization of *Leptotrichia*. The *Leptotrichia* probe sequence was 5’- CACTTCCATTCGGCCCTAATAATC-3’. According to the instructions, the 5-μm thick frozen sections were fixed with 4% (v/v) paraformaldehyde at room temperature for 20min. After fixation, the sections were permeabilized with proteinase K at 37 °C for 20 min and 0.1 M fresh glycine solution for 1 min to stop the proteinase K. After rinsing in PBS buffer, the cells were prehybridized in hybridization buffer at 65 °C for 1 h in a hybridization box. The prehybridization buffer was replaced with a hybridization solution (containing 100 μm Cy3-labeled *Leptotrichia* nucleotide probe), and the sections were incubated at 65 °C for 48 h. The sections were then washed once with 0.2× SSC for 1 min at room temperature, washed three times with formamide plus 4× SSC for 20 min at 65 °C, and five times with PBS for 1 min at room temperature. Finally, the sections were treated with DAPI (Thermo Fisher Scientific) for 5 min. Image acquisition was performed using a Zeiss confocal microscope system (Thornwood). Fluorescence was semiquantitatively assessed on the basis of the mean fluorescence intensity (MFI) of each image.

References

1. Dohlman AB, Arguijo Mendoza D, Ding S, et al. The cancer microbiome atlas: a pan-cancer comparative analysis to distinguish tissue-resident microbiota from contaminants. *Cell Host Microbe.* 2021;29(2):281-298 e285.

2. Cerami E, Gao J, Dogrusoz U, et al. The cBio cancer genomics portal: an open platform for exploring multidimensional cancer genomics data. *Cancer Discov.* 2012;2(5):401-404.

3. Paradis E, Claude J, Strimmer K. APE: Analyses of Phylogenetics and Evolution in R language. *Bioinformatics.* 2004;20(2):289-290.

4. Long X, Wong CC, Tong L, et al. Peptostreptococcus anaerobius promotes colorectal carcinogenesis and modulates tumour immunity. *Nat Microbiol.* 2019;4(12):2319-2330.

|  | Entire cohort (N=153) | |  | T12  (N=61) | |  | T34  (N=92) | |  |
| --- | --- | --- | --- | --- | --- | --- | --- | --- | --- |
|  | Number | % |  | Number | % |  | Number | % | P |
| Age(y) |  |  |  |  |  |  |  |  | 0.308 |
| ≤55 | 59 | 38.6 |  | 23 | 37.7 |  | 36 | 39.1 |  |
| 56-70 | 67 | 43.8 |  | 28 | 45.9 |  | 39 | 42.4 |  |
| >70 | 27 | 17.6 |  | 10 | 16.4 |  | 17 | 18.5 |  |
| Gender |  |  |  |  |  |  |  |  | 0.925 |
| Male | 111 | 72.5 |  | 45 | 73.8 |  | 66 | 71.7 |  |
| Female | 42 | 27.5 |  | 16 | 26.2 |  | 26 | 28.3 |  |
| Stage |  |  |  |  |  |  |  |  |  |
| T12 | 61 | 24.2 |  | 61 | 100.0 |  | 0 | 0.0 |  |
| T34 | 92 | 75.8 |  | 0 | 0.0 |  | 92 | 100.0 |  |
| Smoking status |  |  |  |  |  |  |  |  | 0.179 |
| Nonsmokers | 36 | 23.5 |  | 17 | 27.9 |  | 19 | 20.7 |  |
| Smokers | 115 | 75.2 |  | 44 | 72.1 |  | 71 | 77.2 |  |
| NA | 2 | 1.3 |  | 0 | 0.0 |  | 2 | 2.2 |  |
| Alcohol |  |  |  |  |  |  |  |  | 0.281 |
| Nondrinkers | 40 | 26.1 |  | 15 | 24.6 |  | 25 | 27.2 |  |
| Drinkers | 109 | 71.2 |  | 46 | 75.4 |  | 63 | 68.5 |  |
| NA | 4 | 2.6 |  | 0 | 0.0 |  | 4 | 4.3 |  |
| Race |  |  |  |  |  |  |  |  | 0.344 |
| White | 137 | 89.5 |  | 52 | 85.2 |  | 85 | 92.4 |  |
| African | 12 | 7.8 |  | 7 | 11.5 |  | 5 | 5.4 |  |
| Asian | 2 | 1.3 |  | 1 | 1.6 |  | 1 | 1.1 |  |
| NA | 2 | 1.3 |  | 1 | 1.6 |  | 1 | 1.1 |  |

**Table S1** Demographics and clinical characteristics of the TCGA cohort

Notes: Baseline characteristics between the T12 group and the T34 group were compared with the Pearson χ2 test.

**Table S2** Multivariate Cox Regression Survival Analyses in TCGA cohort

| Variate | P value | Hazard Ratio | 95% confidence interval |
| --- | --- | --- | --- |
| *Leptotrichia* | **0.001** | 0.370 | 0.210-0.653 |
| Age | 0.601 | 1.102 | 0.766-1.584 |
| Gender | 0.485 | 1.214 | 0.704-2.092 |
| Smoke | 0.566 | 1.182 | 0.668-2.093 |
| Alcohol | 0.554 | 1.175 | 0.688-2.008 |
| Race | 0.307 | 0.715 | 0.376-1.361 |
| Stage | 0.918 | 1.029 | 0.595-1.780 |
| Tumor site | 0.253 | 0.825 | 0.593-1.148 |
